# Quiet Quitting Scale: Adaptation and Validation for the Portuguese Nursing Context

**DOI:** 10.3390/nursrep15120411

**Published:** 2025-11-21

**Authors:** João Miguel Almeida Ventura-Silva, Marlene Patrícia Ribeiro, Sónia Cristina da Costa Barros, Susana Filipa Mendes de Castro, Diana Margarida Moreira Sanches, Letícia de Lima Trindade, Paulo João Figueiredo Cabral Teles, Samuel Spiegelberg Zuge, Olga Maria Pimenta Lopes Ribeiro

**Affiliations:** 1 Department of Nursing, Northern Health School of the Portuguese Red Cross, 3720-126 Oliveira de Azeméis, Portugal; 2 RISE-Health, 4200-319 Porto, Portugal; olgaribeiro@esenf.pt; 3 Department of Nursing, Tamega and Sousa Local Health Unit, 4564-007 Penafiel, Portugal; marlene.ribeiro@ulsts.min-saude.pt; 4 Department of Nursing, São João Local Health Unit, 4200-319 Porto, Portugal; sonia.barros@ulssjoao.min-saude.pt; 5 Department of Nursing, Portuguese Oncology Institute of Porto Francisco Gentil, 4200-072 Porto, Portugal; susanafcastro@ipoporto.min-saude.pt; 6 Department of Nursing, Gaia and Espinho Local Health Unit, 4434-502 Vila Nova de Gaia, Portugal; diana.sanches@ulsge.min-saude.pt; 7 Department of Nursing, University of the State of Santa Catarina, Florianópolis 88035-901, Brazil; leticia.trindade@udesc.br; 8 Department of Mathematics and Information Systems, School of Economics and Management, University of Porto, 4200-464 Porto, Portugal; pteles@fep.up.pt; 9 Department of Nursing, Chapecó Region Community University, Chapecó 89809-900, Brazil; samuel.zuge@unochapeco.edu.br; 10 Department of Nursing, Nursing School of Porto, 4200-072 Porto, Portugal

**Keywords:** nursing, occupational stress, psychometrics

## Abstract

Contemporary transformations in the world of work, together with the growing emotional and physical demands in nursing, have led to the emergence of new labor phenomena such as quiet quitting, which reflects changes in professional engagement and in the management of nurses’ well-being. **Objective**: To translate, culturally adapt, and validate the Quiet Quitting Scale for European Portuguese, evaluating its psychometric properties among the nursing population. **Methods**: A cross-sectional validation study was conducted following COSMIN guidelines. The process included forward and back translation, expert panel review, and pretesting with 30 nurses. The psychometric evaluation was carried out with 347 nurses from Northern Portugal. Data were analyzed using descriptive and inferential statistics, internal consistency measures (Cronbach’s α and McDonald’s ω), and confirmatory factor analysis (CFA) with maximum likelihood estimation to assess construct validity. **Results**: The Portuguese version (QQS-PT) maintained the original three-factor structure (Detachment/Disinterest, Lack of Initiative, and Lack of Motivation). The model showed satisfactory fit indices (CFI = 0.936; GFI = 0.901; AGFI = 0.814; TLI = 0.905; RMSEA = 0.133). The overall internal consistency was excellent (α = 0.918; ω = 0.922), with subscale α ranging from 0.788 to 0.924. Composite reliability (CR) ranged from 0.815 to 0.924, and average variance extracted (AVE) from 0.606 to 0.859, confirming convergent and discriminant validity. **Conclusions**: The QQS-PT demonstrated a stable factorial structure, strong reliability, and solid validity evidence. It is a brief and psychometrically sound instrument for assessing quiet quitting among nurses, providing valuable insights for research and management of professional engagement and well-being in healthcare contexts.

## 1. Introduction

In recent decades, technological advances, globalization, and sociocultural transformations have profoundly reshaped the world of work, driving a society in constant adaptation and reconfiguration of values, relationships, and modes of production. These shifts have also altered expectations regarding professional engagement, contributing to emerging forms of psychological withdrawal in organizational settings [1].

In addition, the expansion of digital labor, the focus on productivity, and the adoption of new management models have created important challenges regarding how professionals carry out their activities in the workplace [2]. While these changes can foster organizational innovation and well-being, they also raise tensions related to the work–life balance and the sustainability of labor in healthcare settings [3].

In work environments characterized by high emotional and physical demands, such as nursing, the impact of these transformations becomes particularly evident. Work intensification, combined with constant pressure, direct responsibility for caring for vulnerable individuals, and the increasing complexity of care, exposes professionals to physical and psychological strain [4]. Factors such as long working hours, frequent exposure to suffering and death, staff shortages, and work overload contribute to higher levels of stress, fatigue, and intention to leave the profession [5].

Furthermore, the COVID-19 pandemic exacerbated these conditions, heightening emotional exhaustion and highlighting the urgent need for new adaptive strategies in the work environment. Among nurses, these pressures were even more pronounced, intensifying workload, moral distress, and emotional fatigue, which contributed to shifts in how these professionals managed their engagement and boundaries at work [6,7].

Within this context, a new emerging phenomenon, quiet quitting, has gained attention in healthcare organizations, particularly in nursing. Described as a form of psychological withdrawal or strict contractual adherence, quiet quitting does not involve formal job resignation but rather a redefinition of professional engagement boundaries, in which individuals perform only the tasks stipulated in their job descriptions and refrain from unrecognized or additional efforts [8,9].

Although often associated with professional disengagement, quiet quitting differs from conditions such as burnout, demotivation, or organizational cynicism, as it may represent an intentional strategy to establish healthier limits between professional involvement and personal well-being [10].

Although conceptually related, quiet quitting, burnout, and work disengagement represent distinct constructs that should not be treated interchangeably. Quiet quitting refers to a voluntary and strategic reduction in extra-role effort, in which workers limit their engagement to the tasks formally required, without necessarily experiencing exhaustion [8]. Burnout, in contrast, is a clinically recognized syndrome characterized by emotional exhaustion, depersonalization, and reduced personal accomplishment resulting from chronic work-related stress [11]. Work disengagement involves a progressive decline in psychological investment and organizational commitment, reflecting detachment from professional identity and long-term career goals [12]. Clarifying these distinctions is essential for situating the QQS-PT within the broader theoretical landscape and for specifying that the instrument measures behavioral withdrawal and minimal engagement, rather than emotional exhaustion or organizational detachment.

While quiet quitting may serve as a form of self-regulation, the literature emphasizes that persistent levels of this behavior can lead to progressive detachment from work, resulting in reduced engagement, organizational commitment, and performance. Such effects may ultimately influence the quality and safety of care and the retention of nursing professionals [13,14,15]. Understanding this phenomenon in nursing is therefore particularly relevant, given its potential impact on care safety, professional satisfaction, and team performance.

Despite its growing relevance, research on quiet quitting in healthcare remains scarce, limiting the understanding of the phenomenon and the development of evidence-based organizational policies. To address this gap, Galanis et al. (2023) [15] developed the Quiet Quitting Scale (QQS), consisting of nine items organized into three dimensions—Detachment, Lack of Initiative, and Lack of Motivation—allowing for an objective assessment of work engagement levels.

To date, no validated version of the QQS exists in European Portuguese, specifically adapted to the nursing context in Portugal. It is therefore essential to conduct a rigorous process of translation and cultural adaptation, ensuring semantic, idiomatic, conceptual, and experiential equivalence across items. Such validation may significantly contribute to the advancement of research and to the development of organizational strategies aimed at nurses’ well-being and professional retention [16].

Accordingly, this study aims to translate, culturally adapt, and validate the Quiet Quitting Scale for European Portuguese, evaluating its psychometric properties among the nursing population.

## 2. Materials and Methods

### 2.1. Study Design

This was a cross-sectional study that included the translation, semantic and cultural adaptation of the Quiet Quitting Scale to European Portuguese and the assessment of its psychometric properties among the nursing population. The study followed the recommendations of Streiner and Norman (2018) [17] and was based on the taxonomy, terminology, and definitions of health measurement instruments proposed by the Consensus-based Standards for the Selection of Health Measurement Instruments (COSMIN) [18].

Registered nurses who were present during data collection and engaged in clinical practice were included. Nurses who were absent at the time of data collection, younger than 18 years old, not licensed as registered nurses, with less than six months of professional experience, or working exclusively in administrative or non-clinical roles were excluded, ensuring that the sample represented actively practicing clinical nurses.

### 2.2. Quiet Quitting Scale (QQS)

The QQS, developed by Galanis et al. (2023) [15], is a multidimensional instrument designed to assess the phenomenon of quiet quitting, understood as psychological withdrawal and the intentional limitation of the worker’s engagement to the minimum contractual obligations within an organization. The scale consists of nine items, grouped into three domains: Detachment/Disinterest (four items), Lack of Initiative (three items), and Lack of Motivation (two items).

Responses are recorded on a five-point Likert scale, ranging from 1 (strongly disagree/never) to 5 (strongly agree/always), reflecting the degree of agreement or frequency for each statement. Items 7 to 9 are reverse-scored, requiring recoding before calculating the final score.

The total QQS score is obtained by summing the values of all items and dividing by the total number of valid responses. Higher scores indicate a greater tendency toward quiet quitting. Mean scores can also be calculated for each domain following the same procedure.

### 2.3. Translation and Cross-Cultural Adaptation Processes

The translation and cross-cultural adaptation process of the original version of the QQS into European Portuguese followed the methodological recommendations of Beaton et al. (2000) [19] and the COSMIN guidelines [18], and was structured in the following stages:

1. Forward translation: Conducted independently by two translators who were native speakers of European Portuguese and fluent in Greek. One translator had a background in health sciences, ensuring greater conceptual accuracy, while the other had no training in this area, providing a translation closer to everyday language. This process resulted in two versions (T1 and T2).
2. Synthesis of translations: The preliminary versions (T1 and T2) were compared and jointly analyzed by the translators and the researchers, resulting in a consensus version (TC).
3. Back translation: To verify semantic and conceptual equivalence, two independent translators, native to Greece and fluent in European Portuguese, performed two back translations (BT1 and BT2) of the consensus version (TC) into Greek. The translators were recommended by the Greek Embassy in Portugal and were blinded to the original instrument and the objectives of the study.
4. Expert Committee: Composed of 11 professionals (general care nurses, nurse specialists, nurse managers, nursing faculty members, methodologists, and translators) who evaluated the content validity of the consensus version (TC). All experts held at least a bachelor’s degree and had a minimum of five years of professional experience. The committee received an online form (Microsoft Office Forms) containing the original, T1, T2, and TC versions of each item. The experts assessed semantic, idiomatic, cultural, and conceptual equivalence, as well as clarity and relevance, using a four-point Likert scale: for equivalence, (1) not equivalent, (2) requires major revision, (3) requires minor revision, and (4) equivalent; for clarity, (1) not clear, (2) slightly clear, (3) clear, and (4) very clear; and for relevance, (1) irrelevant, (2) slightly relevant, (3) relevant, and (4) highly relevant. Whenever an item received a score of (1) or (2), the expert was asked to provide reformulation suggestions. Content validity was analyzed using the Content Validity Index (CVI) and the Modified Kappa Coefficient (κ), with acceptable values defined as CVI ≥ 0.90 and κ ≥ 0.75. Suggested revisions were incorporated, and a new review round was conducted until consensus was achieved, resulting in the pre-final version of the instrument.
  In addition, content validation was conducted with a panel of experts selected based on their disciplinary backgrounds, years of professional experience, and bilingual proficiency. The experts assessed the clarity, relevance, and representativeness of the items, allowing for the calculation of the item-level content validity index (I-CVI), the scale-level content validity index (S-CVI), and the modified kappa coefficient. Subsequently, a cognitive debriefing was carried out with a group of nurses during the pilot test, from which qualitative feedback was collected regarding item comprehension, applicability, and potential ambiguities, supporting the final refinements of the instrument.
5. Pre-test: The pre-final version was administered to a sample of 30 nurses to assess the clarity, comprehension, and practical applicability of the items. No difficulties were reported, leading to the approval of the final European Portuguese version of the QQS.

### 2.4. Participants, Setting, Procedure and Validation Process

Following the face validity analysis, the validation phase of the QQS was conducted, during which its psychometric properties were examined.

The sample size was determined based on the recommendations of Comrey and Lee (1992) [20] and Hair et al. (2019) [21], who suggest between 5 and 10 participants per item, with a minimum of 200 cases for factor models. Thus, for the nine items of the QQS, a sample of 45 to 90 participants would have been sufficient; however, a larger sample of 347 nurses was selected to ensure robustness and statistical stability of the results.

A non-probabilistic, purposive sampling method was used. Inclusion criteria were being a nurse, nurse specialist, or nurse manager in Portugal; working in primary or hospital care (Departments of Medicine, Surgery, Women’s and Children’s Health, or Intensive Care and Emergency); and having at least one year of professional experience. Nurses who were absent from work during the data collection period were excluded.

Data collection took place between May and July 2025 in a Local Health Unit in northern Portugal. After obtaining institutional approval, the researchers, in coordination with nurse managers, distributed to participants the Informed Consent Form, the study information sheet, and the questionnaire. Two unmarked envelopes were provided so that participants could return the signed consent form and the completed questionnaire separately, ensuring anonymity.

### 2.5. Data Analysis

All analyses were conducted in R (version 4.x) using the psych, lavaan, and semTools packages. Descriptive and inferential statistics were applied to characterize the sample and assess the psychometric properties of the Portuguese version of the QQS. Missing data were examined prior to the analyses, and no item exceeded 5% of missing responses. Because the proportion of missing values was low and showed a random pattern, they were handled using pairwise deletion. Multivariate outliers were screened through Mahalanobis distance (*p* < 0.001), and no cases met the criteria for exclusion, indicating that the dataset did not present influential multivariate anomalies.

Initially, item homogeneity and internal consistency were evaluated using Cronbach’s alpha (α) and McDonald’s omega (ω) coefficients. The suitability of the correlation matrix for factor analysis was confirmed through the Kaiser–Meyer–Olkin (KMO) index and Bartlett’s test of sphericity. Subsequently, a Confirmatory Factor Analysis (CFA) was performed using maximum likelihood estimation to test the originally proposed three-factor structure (Detachment/Disinterest, Lack of Initiative, and Lack of Motivation).

Model fit was evaluated using conventional criteria: chi-square divided by degrees of freedom (χ^2^/df < 5.0), Comparative Fit Index (CFI ≥ 0.90), Goodness-of-Fit Index (GFI ≥ 0.90), Adjusted Goodness-of-Fit Index (AGFI ≥ 0.80), Tucker–Lewis Index (TLI ≥ 0.90), Root Mean Square Residual (RMR < 0.08), and Root Mean Square Error of Approximation (RMSEA ≤ 0.08, with values ≤ 0.06 indicating good fit). These thresholds follow recommendations from the structural equation modeling literature.

Composite reliability (CR) was calculated for each domain, while Average Variance Extracted (AVE) was used to verify convergent validity. Discriminant validity was confirmed when the AVE for each dimension exceeded the squared correlations between the factors. Prior to the CFA, the data were assessed for multivariate normality, outliers, and absence of multicollinearity, ensuring that statistical assumptions were met. Multivariate normality was assessed using Mardia’s coefficient, and the results indicated acceptable levels of skewness and kurtosis for CFA.

### 2.6. Reliability, Validity and Bias Control

Reliability was assessed through internal consistency (α, ω) and composite reliability (CR). Evidence of validity included content validity, construct validity (through CFA), convergent validity, and discriminant validity. To minimize potential biases, data collection was conducted under standardized conditions, ensuring anonymity and the absence of any hierarchical relationship between researchers and participants.

### 2.7. Ethical Procedures

The study was approved by the Ethics Committee of the Unidade Local de Saúde São João, where the research was conducted (Approval No. 51-2025). All participants were informed about the study objectives and procedures and signed the Informed Consent Form prior to participation. Data collection was voluntary and anonymous; participants sealed the consent form and the questionnaire in separate, unmarked envelopes to ensure confidentiality.

## 3. Results

The sample consisted of 347 nurses, predominantly female (85.9%), with a mean age of 41.7 ± 9.8 years (ranging from 23 to 65 years). The majority were married or in a domestic partnership (59.7%) and held a graduation degree (81.8%). Regarding professional category, 65.4% were nurses and 31.7% were nurse specialists, with Rehabilitation Nursing being the most represented specialty (34.5%). In terms of work setting, there was a predominance of hospital care, particularly in medical departments (30.0%). The average professional experience was 18.5 ± 9.7 years among nurses and 8.6 ± 6.0 years among specialists, with an average of 11.6 ± 9.0 years working in their current department (Table 1).

The analysis of homogeneity and internal consistency showed that the QQS presented inter-item correlations ranging from 0.362 to 0.860 (mean = 0.549) and corrected item–total correlations ranging from 0.530 to 0.824 (mean = 0.710), indicating adequate coherence among items. The overall Cronbach’s alpha coefficient was 0.918, and McDonald’s omega was 0.922, demonstrating excellent internal consistency.

Each dimension also showed good reliability indicators: Detachment/Disinterest (α = 0.880; ω = 0.900), Lack of Initiative (α = 0.788; ω = 0.852), and Lack of Motivation (α = 0.924). The mean item–total correlation was high (0.710), and the Guttman split-half (0.819) and Spearman–Brown (0.849) coefficients further supported the internal stability of the scale (Table 2 and Table 3).

**Figure 1:**
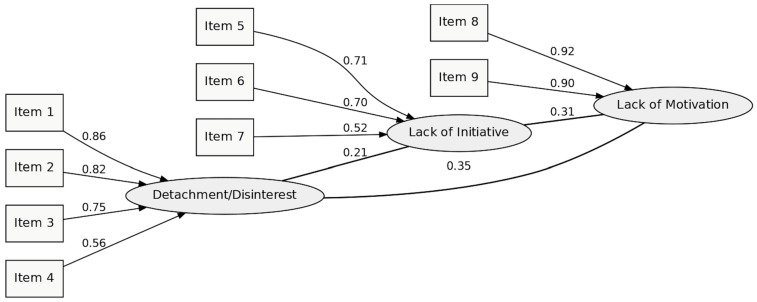
Confirmatory three-factor model of the Quiet Quitting Scale (QQS-PT).

The Kaiser–Meyer–Olkin (KMO) measure of sampling adequacy was 0.869, exceeding the COSMIN benchmark for meritorious adequacy (>0.80). Bartlett’s test of sphericity was significant (*p* < 0.001), confirming that the correlation matrix was suitable for factor analysis (Table 3). In the confirmatory factor analysis, the proposed three-factor model—Detachment/Disinterest, Lack of Initiative, and Lack of Motivation—showed satisfactory overall fit after correlating error terms with modification indices greater than 11. The main fit indices indicated good model quality: CFI = 0.936; GFI = 0.901; AGFI = 0.814; IFI = 0.937; TLI = 0.905; RMR = 0.068; RMSEA = 0.133; χ^2^/df = 7.144. Approximately 70% of the nonredundant residuals showed absolute values below 0.05, supporting the good fit of the model (Table 4; Figure 1). Modification indices were inspected, and only theoretically justified residual correlations were retained, avoiding data-driven adjustments.

The standardized factor loadings ranged from 0.524 to 0.924, indicating that most items showed high loadings on their respective dimensions (Figure 1). Individual item reliability ranged from 0.275 to 0.865, suggesting that most items demonstrated strong factorial representation within their corresponding factors.

Composite reliability values were 0.887 for Detachment/Disinterest, 0.815 for Lack of Initiative, and 0.924 for Lack of Motivation, all above the recommended threshold of 0.70, confirming the internal stability of each construct. The squared correlations between the three factors (r^2^ = 0.757, 0.601, and 0.680) were lower than their respective AVE values, ensuring discriminant validity (Figure 1).

Convergent validity was confirmed through the Average Variance Extracted (AVE), with values of 0.667 for Detachment/Disinterest, 0.606 for Lack of Initiative, and 0.859 for Lack of Motivation, all exceeding the recommended threshold of 0.50. Discriminant validity was also supported, as the AVE values for each dimension were greater than the squared correlations between factors, which ranged from 0.601 to 0.757 (Table 3).

In summary, the Portuguese version of the Quiet Quitting Scale demonstrated a stable factorial structure, high internal consistency, and strong evidence of convergent and discriminant validity. The overall psychometric results confirm the adequacy of the scale for assessing the quiet quitting phenomenon in the nursing context.

## 4. Discussion

The validity and reliability of the Portuguese version of the Quiet Quitting Scale (QQS-PT) for the nursing context were confirmed through the results of this study. The original three-factor structure—comprising the dimensions Detachment/Disinterest, Lack of Initiative, and Lack of Motivation [15], was replicated, showing adequate fit indices and high internal consistency. These findings reinforce the cross-cultural stability of the instrument and its applicability in different professional contexts [22,23].

Cross-cultural findings further support the relevance of validating the QQS in Portugal. The Turkish adaptation demonstrated good reliability among healthcare workers, confirming the scale’s applicability in a non-Western context [24]. Similarly, the Greek validation reported a stable three-factor structure and empirical cut-off points, offering one of the first European benchmarks for quiet quitting [15]. These results provide useful reference points for interpreting the present findings and highlight both convergences and cultural nuances in how quiet quitting manifests among nursing professionals.

The adequacy of the sample for factor analysis was confirmed by the KMO values and Bartlett’s test of sphericity, demonstrating the factorability of the correlation matrix. In the confirmatory factor analysis, the main fit indices, such as CFI, TLI, and GFI, showed good values, confirming that the theoretical model fits the empirical data well [21].

Although the RMSEA presented a borderline value, this result is acceptable for models with complex structures and a small number of items [21]. This pattern has also been reported in other short psychometric instruments, in which limited degrees of freedom tend to inflate RMSEA values.

However, it is important to acknowledge that the elevated RMSEA indicates potential model strain, likely related to the restricted degrees of freedom inherent in short scales. Such values warrant cautious interpretation and reinforce the need for future studies to test the structure in broader and more heterogeneous samples.

The overall internal consistency of the scale was excellent, with Cronbach’s α and McDonald’s ω coefficients above 0.90, and composite reliability (CR) values ranging from 0.815 to 0.924. These indicators are higher than those obtained in the original study (overall α = 0.803) [15], indicating that, in the Portuguese context, the items displayed greater internal homogeneity.

These results demonstrate the psychometric robustness of the QQS-PT and confirm that the three dimensions maintain strong conceptual coherence among themselves. In addition, the clearer association between items and their respective latent factors suggests that the scale performs consistently in the Portuguese nursing context, reinforcing its suitability for assessing different manifestations of quiet quitting among professionals [25].

Convergent and discriminant validity were also confirmed, with AVE values above 0.50 for all dimensions and higher than the squared inter-factor correlations. These results support the theoretical distinction among the factors, showing that Detachment/Disinterest, Lack of Initiative, and Lack of Motivation represent independent, though interrelated, dimensions of the quiet quitting phenomenon [23,26].

When comparing the results of this study with those of Galanis et al. (2023) [15], convergence is observed both in the factorial structure and in the magnitude of factor loadings. However, applying the scale to a specific population of nurses adds additional value, as this professional group faces working conditions characterized by high emotional, physical, and moral demands [27]. Therefore, understanding quiet quitting in this context is essential for interpreting forms of disengagement that may reflect an adaptive response to exhaustion or a gradual decline in professional involvement.

It should also be noted that the sample predominantly represented nurses from a single region of Portugal and was mostly composed of women, which may influence how quiet quitting behaviors are expressed and interpreted. These demographic characteristics should be considered when generalizing the findings to other nursing contexts.

The literature indicates that quiet quitting may emerge as a self-preservation mechanism in response to work overload and emotional exhaustion [28]. From this perspective, limiting engagement to strictly contractual activities may represent an attempt to maintain a balance between personal and professional life. However, high and prolonged levels of this behavior may compromise organizational commitment and the quality of care provided [29,30].

The results obtained in this study suggest that the QQS-PT is sufficiently sensitive to capture these nuances, contributing to the early detection of signs of occupational withdrawal. Previous research in nursing has shown that psychological withdrawal behaviors, such as reduced initiative, minimal task engagement, and strict adherence to basic job duties, are associated with negative outcomes, including higher levels of emotional exhaustion, increased intention to leave the profession, and reduced quality of care. These findings reinforce the importance of identifying early indicators of disengagement among nurses, as timely recognition may help prevent more serious consequences for both workers and healthcare systems [25].

From a theoretical standpoint, the results can be interpreted through the lens of motivational and organizational models. According to Social Exchange Theory, when employees perceive that they are not receiving adequate recognition or reward for their effort, a decrease in engagement or psychological withdrawal is likely to occur [31,32]. These results demonstrate the psychometric robustness of the QQS-PT and confirm that the three dimensions maintain strong conceptual coherence among themselves [12,33]. In addition, the clearer association between items and their respective latent factors suggests that the scale performs consistently in the Portuguese nursing context, reinforcing its suitability for assessing different manifestations of quiet quitting among professionals [10,22,23].

The integration of these theoretical perspectives reinforces the interpretation of quiet quitting as a multidimensional construct influenced not only by individual motivation but also by organizational culture, leadership style, and perceptions of fairness [22], potentially serving as a significant predictor of quiet quitting behavior among healthcare professionals [9].

Furthermore, the absence of measurement invariance testing across demographic subgroups limits the ability to determine whether the scale functions equivalently among nurses with different characteristics, such as age, gender, or years of experience. This remains an important direction for future validation efforts.

Therefore, it is recommended that the scale be applied in different practice contexts (primary care, long-term care, and education) and that longitudinal analyses be conducted to assess the temporal stability of the instrument and its predictive validity regarding variables such as burnout, engagement, and turnover intention.

From a comparative perspective, the Portuguese results reveal psychometric patterns similar to those observed in other cultural contexts, suggesting that the quiet quitting phenomenon may display universal characteristics, although modulated by cultural and organizational factors [34]. Short cross-cultural evidence has also supported the instrument’s applicability. The Turkish adaptation demonstrated good reliability among healthcare workers, while the Greek validation confirmed the stability of the three-factor structure. These findings offer useful comparative benchmarks for the present results.

In practical terms, the results have relevant implications for healthcare organizations. The QQS-PT constitutes an accessible instrument for monitoring nurses’ engagement and identifying early signs of detachment. Nursing managers and leaders can use it in periodic assessments to guide preventive interventions, such as recognition programs, mentoring strategies, participatory leadership models, and well-being initiatives [35]. Systematic application of the QQS-PT can thus contribute to creating organizational climates that value professional recognition, emotional support, and work–life balance.

The discussion of practical applications also highlights the potential of the QQS-PT to support health workforce management and planning policies. By quantifying levels of quiet quitting within institutions, decision-makers can obtain empirical data to guide retention strategies, improve working conditions, and prevent the worsening of professional disengagement—a critical issue in light of the global nursing shortage [36].

From a practical standpoint, the QQS-PT provides healthcare institutions with a useful tool for identifying early signs of psychological withdrawal among nurses. Detecting patterns such as reduced initiative or minimal engagement may support timely actions to improve working conditions and prevent disengagement-related outcomes. Its systematic use may also help reduce turnover intention and promote workforce stability, contributing to safer and more sustainable nursing care.

Among the limitations of this study, the use of a convenience sample composed mostly of women from a single region of the country may limit the generalization of the findings. Additionally, the study did not assess temporal stability through test–retest reliability, which would allow verification of the instrument’s stability over time. Concurrent validity was also not examined, as no gold-standard instrument exists to evaluate quiet quitting, limiting the possibility of comparing the QQS-PT with an external criterion. Moreover, it is noteworthy that one of the limitations was that measurement invariance across subgroups (e.g., age, gender, years of experience, or type of institution) was not tested.

Despite these limitations, the high internal consistency and acceptable model fit indices indicate that the QQS-PT is a valid and reliable instrument for assessing quiet quitting among nursing professionals. Future multicenter studies should include different regions of the country, private healthcare institutions, and long-term care units and incorporate additional psychometric tests, such as test–retest reliability, concurrent validity, and measurement invariance, to further strengthen the external validity and robustness of the instrument.

The validation of the QQS-PT represents a significant advancement for nursing research and practice, providing a concise, reliable, and conceptually sound measure for assessing quiet quitting. The systematic use of this instrument can contribute to the early diagnosis of signs of demotivation and to the implementation of policies aimed at professional appreciation and the promotion of nurses’ mental health, fostering more sustainable and humanized work environments.

## 5. Conclusions

The Portuguese version of the Quiet Quitting Scale (QQS-PT) demonstrated robust psychometric properties, confirming its validity and reliability for assessing the phenomenon of quiet quitting among nurses. The instrument retained the original three-factor structure—Detachment/Disinterest, Lack of Initiative, and Lack of Motivation—with satisfactory model fit indices, high internal consistency, and strong evidence of convergent and discriminant validity.

The QQS-PT provides a brief, conceptually coherent, and psychometrically sound tool for evaluating the psychological withdrawal and behavioral disengagement that characterize quiet quitting in healthcare settings. By enabling the assessment of this phenomenon among nursing professionals, the scale contributes to a more accurate understanding of the relationship between work engagement, well-being, and professional retention.

Future research should further test the QQS-PT in larger and more diverse samples, exploring its predictive validity and cross-cultural stability. The systematic use of this instrument can assist healthcare organizations and policymakers in identifying early indicators of disengagement, promoting supportive strategies, and fostering healthier, more sustainable work environments for nurses.

## Figures and Tables

**Table 1:** Sociodemographic, academic, and professional characteristics of the nursing sample (N = 347).

Variable	
Gender	n (%)
Male	49 (14.1)
Female	298 (85.9)
Age	years
Mean (±SD)	41.7 (±9.8)
Minimum; maximum	23; 65
Marital status	n (%)
Not married	113 (32.6)
Married/nonmarital partnership	207 (59.7)
Divorced	24 (6.9)
Widower	3 (0.9)
Educational qualification	n (%)
Graduation	284 (81.8)
Master’s degree	63 (18.2)
Work context	n (%)
Primary health care unit	35 (10.1)
Hospital healthcare, department of medicine service	104 (30.0)
Hospital healthcare, department of surgery service	90 (25.9)
Hospital healthcare, department of intensive care medicine	53 (15.3)
Hospital healthcare, department of women and children	65 (18.7)
Condition of exercise of the profession	n (%)
Nurse	227 (65.4)
Specialist nurse	110 (31.7)
Manager nurse	10 (2.9)
Specialty area	n (%)
Medical-surgical nursing	29 (26.4)
Rehabilitation nursing	38 (34.5)
Maternal and obstetric health nursing	10 (9.1)
Pediatric and child health nursing	20 (18.2)
Community nursing	13 (11.8)
Time of professional experience in the profession	years
Mean (±SD)	18.5 (±9.7)
Minimum; maximum	1; 42
Time of professional practice in the current service	years
Mean (±SD)	11.6 (±9.0)
Minimum; maximum	1; 42
Time of professional practice as a specialist nurse	years
Mean (±SD)	8.6 (±6.0)
Minimum; maximum	1; 29
Time of professional practice as a manager nurse	years
Mean (±SD)	15.5 (±2.5)
Minimum; maximum	12; 20

Note: SD = Standard deviation; n = absolute frequency; % = relative frequency.

**Table 2:** Analysis of homogeneity and internal consistency of the Portuguese version of the QQS.

Parameter	Value
Inter-item correlation (minimum–maximum)	0.362–0.860
Inter-item correlation (mean)	0.549
Corrected item-total correlation (minimum–maximum)	0.530–0.824
Item-toral correlation (mean)	0.710
Cronbach’s alpha (global)	0.918
McDonald’s omega (global)	0.922
Guttman split-half	0.819
Spearman–Brown	0.849

**Table 3:** Descriptive statistics, corrected item–total correlations, internal consistency indices (Cronbach’s α and McDonald’s ω), and construct reliability (CR and AVE) for the nine items and three factors of the Portuguese version of the Quiet Quitting Scale (QQS-PT) (N = 347).

Factor/Item	Mean (SD)	Corrected Item-Total Correlation	KMO	Cronbach’s α McDonald’s ω	Cronbach’s α If Item Deleted	Composite Reliability (CR)	Average Variance Extracted (AVE)
Detachment/Disinterest	2.49 (0.91)			0.88 0.90		0.88	0.67
1. Estou apenas a cumprir os requisitos mínimos do meu trabalho, para não falhar, sem contribuir com coisas extra	2.43 (0.88)	0.82	0.92	--	0.91	--	--
2. Se um colega pode fazer parte do meu trabalho, então deixo que seja ele a fazê-lo	2.61 (1.02)	0.75	0.88	--	0.91	--	--
3. Faço o máximo de pausas possíveis e mais longas	2.54 (0.96)	0.76	0.91	--	0.91	--	--
4. Com que frequência finge já estar a fazer uma atividade/tarefa para evitar que lhe seja atribuída outra?	2.37 (0.91)	0.60	0.88	--	0.91	--	--
Lack of Initiative	2.85 (0.86)			0.85 0.85		0.81	0.61
5. Não expresso opiniões e ideias sobre o meu trabalho porque temo que me possa ser atribuído mais trabalho	2.72 (0.93)	0.81	0.88	--	0.91	--	--
6. Não expresso opiniões e ideias sobre o meu trabalho, porque acredito que nada irá mudar	2.88 (0.82)	0.76	0.87	--	0.91	--	--
7. Com que frequência toma a iniciativa no seu trabalho?	2.94 (0.87)	0.53	0.88	--	0.91	--	--
Lack of Motivation	3.27 (0.74)	--	--	0.92 0.92	--	0.86	0.85
8. Sinto-me motivado no meu trabalho	3.21 (0.78)	0.66	0.79	--	0.92	--	--
9. Sinto-me entusiasmado quando trabalho	3.33 (0.73)	0.68	0.80	--	0.92	--	--

Note: SD = Standard deviation; α = Cronbach’s alpha; ω = McDonald’s omega; CR = Composite reliability; AVE = Average variance extracted. All factors demonstrated satisfactory internal consistency (α and ω > 0.70), high composite reliability (CR > 0.70), and adequate convergent validity (AVE > 0.50).

**Table 4:** Fit indices of the three-factor model of the Quiet Quitting Scale (CFA, N = 347).

Index	Value	Interpretation
CFI	0.936	Good fit
GFI	0.901	Good fit
AGFI	0.814	Acceptable
IFI	0.937	Good fit
TLI	0.905	Good fit
RMR	0.068	Adequate
RMSEA	0.133	Borderline, close to acceptability
MECVI	0.621	Stable
χ^2^/df	7.144	Within the expected range for complex models

Note: CFA = Confirmatory Factor Analysis; CFI = Comparative Fit Index; GFI = Goodness of Fit Index; AGFI = Adjusted Goodness of Fit Index; IFI = Incremental Fit Index; TLI = Tucker–Lewis Index; RMR = Root Mean Square Residual; RMSEA = Root Mean Square Error of Approximation; MECVI = Modified Expected Cross-Validation Index.

## Data Availability

The data presented in this study is available on request from the corresponding author. The data are not publicly available due to ethical restrictions.

## Public Involvement Statement

No public involvement in any aspect of this research.

## Guidelines and Standards Statement

This manuscript was prepared on the basis of COSMIN for methodological study research.
